# Species conservation profile of the stenoendemic cave spider *Pimoa delphinica* (Araneae, Pimoidae) from the Varaita valley (NW-Italy)

**DOI:** 10.3897/BDJ.5.e11509

**Published:** 2017-01-19

**Authors:** Stefano Mammola, Gustavo Hormiga, Marco Isaia

**Affiliations:** 1 University of Turin, Department of Life Sciences and Systems Biology, Torino, Italy; 2 IUCN SSC Spider & Scorpion Specialist Group, Torino, Italy; 3 The George Washington University, Washington DC, United States of America

**Keywords:** Western Alps, IUCN, Cave, Military bunker, red list, troglophile species

## Species information

Scientific name: Pimoa delphinica

Species authority: Mammola, Hormiga & Isaia, 2016

Kingdom: Animalia

Phylum: Arthropoda

Class: Arachnida

Order: Araneae

Family: Pimoidae

Taxonomic notes: Pimoa delphinica is a medium-sized spider (cephalothorax: ca. 4 mm, abdomen: ca. 7 mm), with slender legs and a brown-reddish coloration (Fig. 1). The species—previously misidentified with P. rupicola (Simon, 1884)—was described in 2016. It is readily distinguishable from the other species of European pimoids by morphological characters in male and female genitalia (cf. Mammola et al. 2016b).

Region for assessment: Global

## Geographic range

Biogeographic realm: Palearctic

Countries: Italy

**Figure 2.**
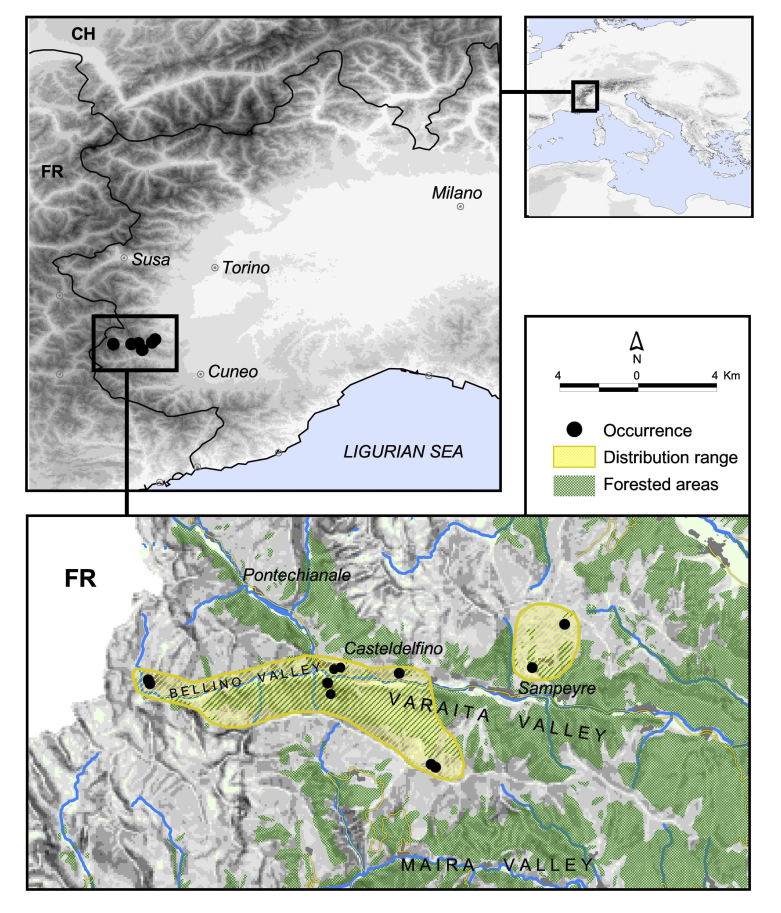
Known distribution of *Pimoa delphinica*.

Map of records (image): Fig. 2

Map of records (Google Earth): Suppl. material 1

Basis of EOO and AOO: Known habitat extent

Basis (narrative): During relatively intense speleological research studies in the Western Alps, we collected Pimoa delphinica in a few caves and military bunkers in the municipalities of Casteldelfino, Sampeyre and Sant'Anna di Bellino (Varaita valley, Western Alps, Province of Cuneo, Italy). Specifically, the species occurred in four small wild caves and a few subterranean military bunkers in Casteldelfino and Sant'Anna di Bellino and an abandoned house in the hamlet of Becetto (Sampeyre). Two additional localities reported in Isaia et al. (2011) and Mammola et al. (2016b)—Buco del Nebin 1 [Speleological Cadastre: Pi 1158] and Buco del Nebin 2 [Pi 1159] caves—are in need of verification, since only juvenile specimens were collected. However, even if confirmed, these additional localitites would fall within the known extent of occurrence of the species (cf. Suppl. material 1). Despite our extensive study of Pimoa specimens from Italy and France—including material stored in Museums or private collections and original records—we have not been able to find additional records of Pimoa delphinica outside this small area (detail in Mammola et al. 2016b).We used the verified occurrence records of the species to calculate the extent of occurrence (EOO) as the minimum convex hull and the area of occupancy (AOO) through a 2x2 km2 grid, as implemented in the red R package (Cardoso 2016).

Min Elevation/Depth (m): 1230

Max Elevation/Depth (m): 2242

Range description: Pimoa delphinica is a stenoendemic species with a restricted Western Alpine distribution. The species is recorded in seven nearby localities, in the high Varaita valley. Its distribution range represents a small enclave (ca. 20 km2) within the range of distribution of the sister species P. graphitica Mammola, Hormiga & Isaia, 2016.

## New occurrences

### Materials

- **Type status:** Other material. **Occurrence:** recordedBy: Stefano Mammola, Alessandro Girodo; individualCount: 2; sex: female; lifeStage: adult, juvenile; **Taxon:** scientificName: Pimoa delphinica; kingdom: Animalia; phylum: Arthropoda; class: Arachnida; order: Araneae; family: Pimoidae; genus: Pimoa; specificEpithet: delphinica; scientificNameAuthorship: Mammola, Hormiga, Isaia, 2016; **Location:** country: Italy; stateProvince: Piedmont; county: CN; municipality: Casteldelfino; locality: Military bunker 1 near the road to Casteldelfino; verbatimElevation: 1280 m; minimumElevationInMeters: 1280; maximumElevationInMeters: 1280; locationRemarks: Abandoned military bunker (II World War); verbatimCoordinates: 44°35'07.21"N, 7°04'40.02"E; georeferenceProtocol: GPS; **Identification:** identifiedBy: Stefano Mammola, Marco Isaia; dateIdentified: 2016; **Event:** samplingProtocol: hand collected; eventDate: 12 Dec 2016; **Record Level:** basisOfRecord: PreservedSpecimen
- **Type status:** Other material. **Occurrence:** recordedBy: Stefano Mammola, Alessandro Girodo; individualCount: 3; sex: female; lifeStage: adult, juvenile; **Taxon:** scientificName: Pimoa delphinica; kingdom: Animalia; phylum: Arthropoda; class: Arachnida; order: Araneae; family: Pimoidae; genus: Pimoa; specificEpithet: delphinica; scientificNameAuthorship: Mammola, Hormiga, Isaia, 2016; **Location:** country: Italy; stateProvince: Piedmont; county: CN; municipality: Casteldelfino; locality: Military bunker 2 near the road to Casteldelfino; verbatimElevation: 1288 m; minimumElevationInMeters: 1288; maximumElevationInMeters: 1288; locationRemarks: Abandoned military bunker (II World War); verbatimCoordinates: 44°35'07.70"N, 7°04'40.15"E; georeferenceProtocol: GPS; **Identification:** identifiedBy: Stefano Mammola, Marco Isaia; dateIdentified: 2016; **Event:** samplingProtocol: hand collected; eventDate: 12 Dec 2016; **Record Level:** basisOfRecord: PreservedSpecimen
- **Type status:** Other material. **Occurrence:** recordedBy: Alessandro Girodo, Paolo Bertacco; individualCount: 2; sex: females; lifeStage: adults; **Taxon:** scientificName: Pimoa delphinica; kingdom: Animalia; phylum: Arthropoda; class: Arachnida; order: Araneae; family: Pimoidae; genus: Pimoa; specificEpithet: delphinica; scientificNameAuthorship: Mammola, Hormiga, Isaia, 2016; **Location:** country: Italy; stateProvince: Piedmont; county: CN; municipality: Casteldelfino; locality: Miniera di Casteldelfino, galleria Auriol (Borgata Ciampanesio); verbatimElevation: ca. 970 m; locationRemarks: Mineshaft; verbatimCoordinates: 44°35'10.3"N, 7°07'06.6"E; verbatimCoordinateSystem: WGS84; decimalLatitude: 44.5862; decimalLongitude: 7.1185; georeferenceProtocol: GPS; **Identification:** identifiedBy: Stefano Mammola, Marco Isaia; dateIdentified: 2016; **Event:** samplingProtocol: hand collected; eventDate: 16 Dec 2016; habitat: Subterranean; **Record Level:** basisOfRecord: PreservedSpecimen

## Extent of occurrence

EOO (km2): 26

Trend: Unknown

Justification for trend: The species is troglophile (sensu Sket 2008), showing a preference for dark and moisty habitats. Yet, the species exhibits a moderate plasticity in its ecological requirements, being able to colonize both cave and extra-cave environments, such as forests in high altitude habitats. The distribution range of Pimoa delphinica is enclosed within the range of the more widespread P. graphitica, which is apparently competing with the former thus limiting its expansion (Mammola et al. 2016b). The subterranean habitats colonized by P. delphinica are as yet not threatened by direct human activities. However, biogeographic studies and genetic data suggested that past climate change determined strong contraction in the distribution ranges of the species of alpine Pimoa (Mammola et al. 2016b, Mammola et al. 2015). It is thus plausible that anthropogenic climate change may determine reduction or habitat shift for this species. In order to confirm this hypothesis statistically, a deeper study of the detailed occurrence of this species in its distribution range is required.

Causes ceased?: Unknown

Causes understood?: Unknown

Causes reversible?: Unknown

Extreme fluctuations?: No

## Area of occupancy

Trend: Unknown

Justification for trend: See paragraph "Extent of Occurrence".

Causes ceased?: Unknown

Causes understood?: Unknown

Causes reversible?: Unknown

Extreme fluctuations?: No

AOO (km2): 12

## Locations

Number of locations: 

Trend: Stable

Extreme fluctuations?: No

## Population

Number of individuals: Unknown.

Trend: Unknown

Justification for trend: No information about population size are currently available.

Causes ceased?: Unknown

Causes understood?: Unknown

Causes reversible?: Unknown

Extreme fluctuations?: No

Population Information (Narrative): A census of the population has never been attempted. According to our observations, populations are locally abundant. In two caves in which Pimoa delphinica was found in syntopy with the congeneric P. graphitica, mixed nuclear alleles between the two species have been found, indicating the existence of unidirectional introgression of males of P. graphitica into females of P. delphinica (Mammola et al. 2016b).

## Subpopulations

Number of subpopulations: 2

Trend: Stable

Justification for trend: Examining the known range of distribution and taking into account habitat connectivity, it is possible to identify two subpopulations within the range. The first subpopulation includes the localitites from Casteldelfino and the Bellino valley, which are more connected through alpine scree and larch woods, acting as potential route of dispersal. The other subpopulation is found on the other slope of the Varaita valley, in the nearby of the hamlet of Becetto (municipality of Sampeyre). The subpopulation are as yet not threatened.

Extreme fluctuations?: No

Severe fragmentation?: No

Justification for fragmentation: 

## Habitat

System: Terrestrial

Habitat specialist: Yes

Habitat (narrative): The species primarily lives in the twilight zone of wild caves and other similar sheltered habitats, in high alpine environments (Fig. 3a). Healthy populations were also observed in artificial subterranean habitat (military bunkers and mines), offering suitable cool climatic conditions (Fig. 3b, c, d). Further individuals of P. delphinica were collected in pitfall traps placed within the rocky debris on the floor of the cellar of an abandoned cottage (Becetto, Sampeyre). During summertime, we observed juveniles—tentatively classified as P. delphinica—in an epigean environment, near the locus typicus (Fig. 3c), in a larch (Larix decidua) wood. Accordingly, it is possible that juveniles of P. delphinica may be able to disperse trough epigean habitats under suitable climatic condition. Extra-cave dispersal was also documented for the congeneric alpine species P. graphitica and P. rupicola, as justified by occasional catches of juveniles and males in pitfall traps placed in the leaf litter of broad-leaved woods at mid-altitudes (e.g., Isaia et al. 2015, Isaia et al. 2014, Jackson 1929, Mammola et al. 2015, Mammola et al. 2016b).

Trend in extent, area or quality?: Stable

### Habitat

Habitat importance: Major Importance

Habitats: 7. Caves and Subterranean Habitats (non-aquatic)

### Habitat

Habitat importance: Marginal

Habitats: 1. Forest

## Ecology

Size: Total length (leg excluded) = Male: 7 mm, Female: 10.5 mm

Generation length (yr): 1

Dependency of single sp?: No

Ecology and traits (narrative): Little is known about the ecology of Pimoa delphinica. We report the result of our sporadic observations, which are not supported by specific studies or statistical inference. Like other Pimoa species (cf. Mammola et al. 2016a, Mammola et al. 2015, Hormiga 1994), P. delphinica exhibits a moderate ecological plasticity. In caves, it is found preferentially in the twilight zone. We observed adult males and females during the summertime. Adults display thanatotic behaviour when disturbed, possibly as a protection against predators (cf. Rogers and Simpson 2014, Novak et al. 2016). Depositions of cocoons occurs in July and cocoons are guarded by females. Females affix substrate particles to their cocoons (Mammola et al. 2016b).

## Threats

Justification for threats: See "Extent of occurrence".

### Threats

Threat type: Future

Threats: 11.1. Climate change & severe weather - Habitat shifting & alteration

## Conservation

Justification for conservation actions: A portion of the distribution range of Pimoa delphinica falls within the border of the Natural Park "Parco del Monviso".The installation of information panels educating the visitors about this peculiar endemic species would positively increase the awareness of the caves as a natural heritage deserving protection.

### Conservation actions

Conservation action type: In Place

Conservation actions: 2.1. Land/water management - Site/area management

### Conservation actions

Conservation action type: Needed

Conservation actions: 4. Education & awareness

## Other

### Research needed

Research needed: 1. Research

Justification for research needed: 

## Supplementary Material

Supplementary material 1 Extent of Occurrence of *Pimoa delphinica* Data type: Geographic range
File: oo_102120.kml Mammola S., Hormiga G., Isaia M.

## Figures and Tables

**Figure 1a.**
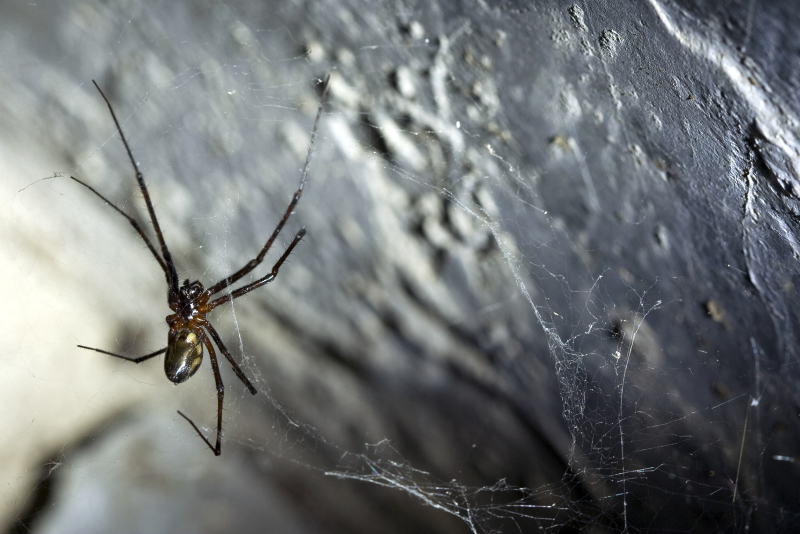
Ventral habitus.

**Figure 1b.**
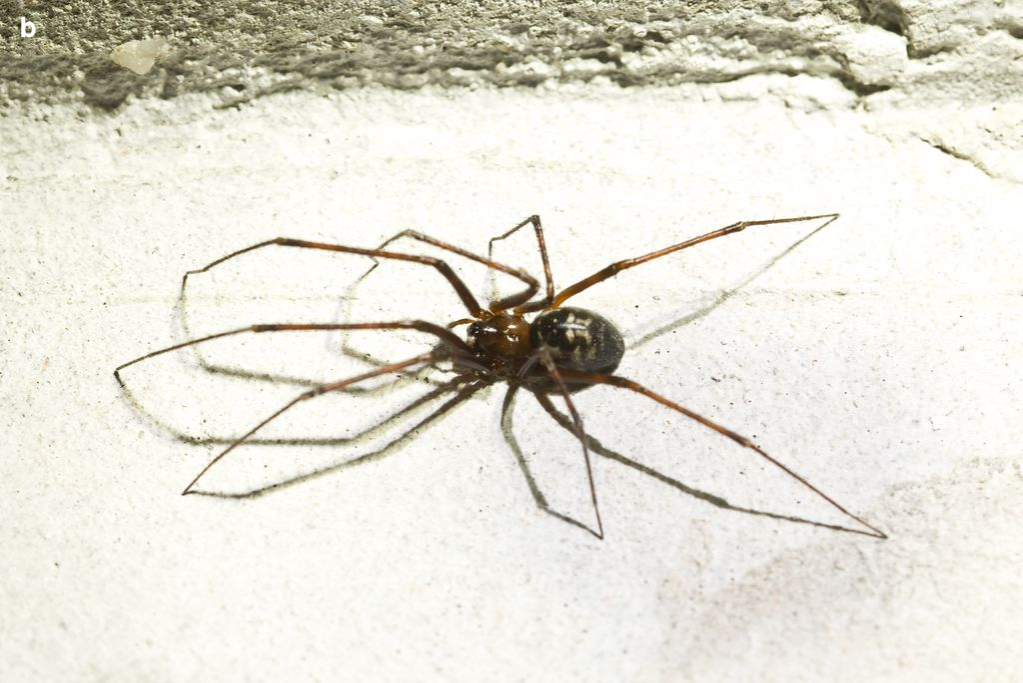
Dorsal habitus.

**Figure 3a.**
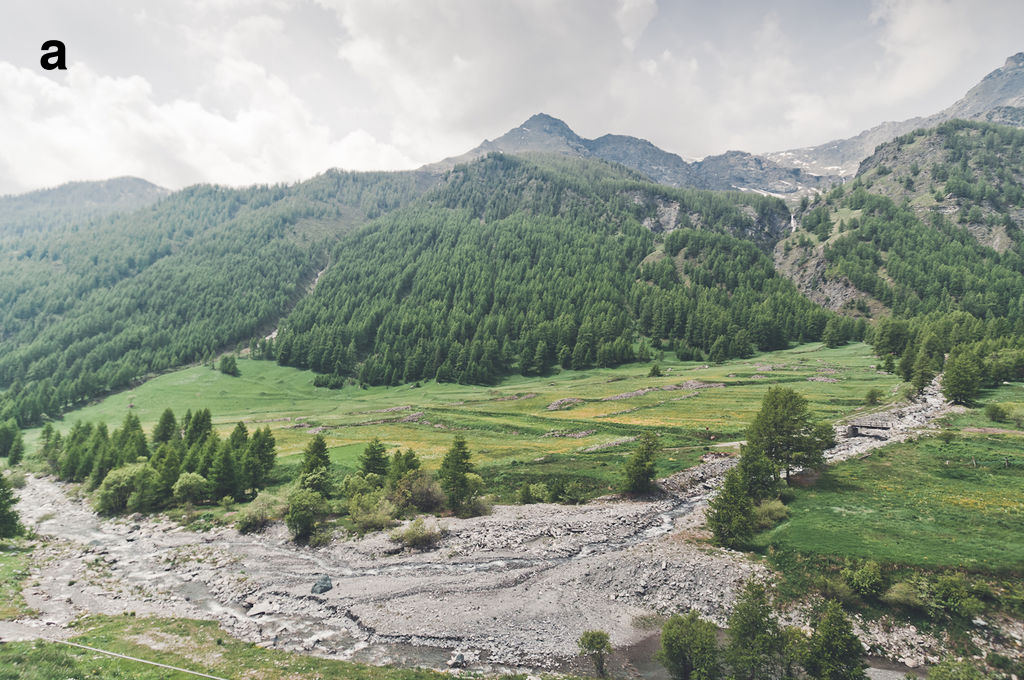
The typical alpine environment in the high Varaita valley where the caves colonized by *Pimoa delphinica* are found.

**Figure 3b.**
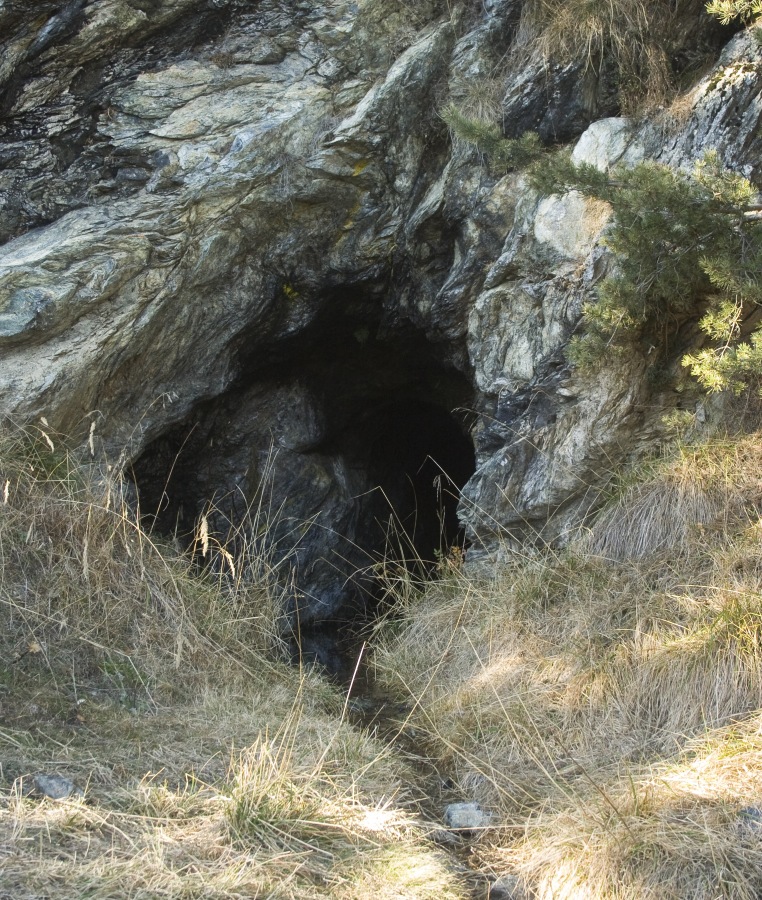
An abandoned mineshaft colonized by the species.

**Figure 3c.**
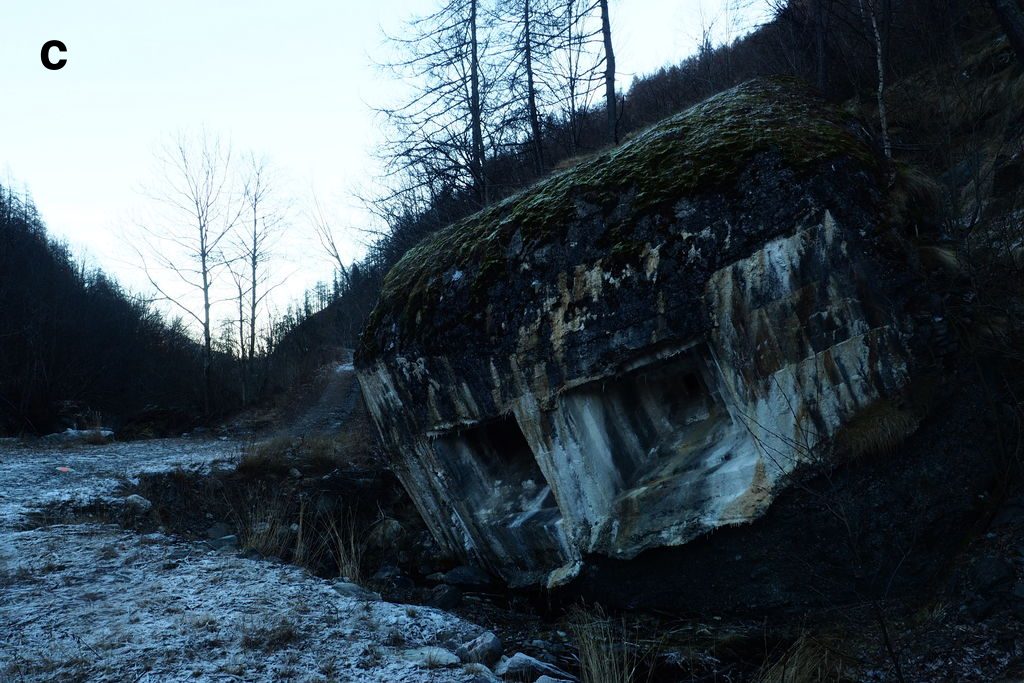
A disused military bunkers from the Second World War, *locus typicus* of the species.

**Figure 3d.**
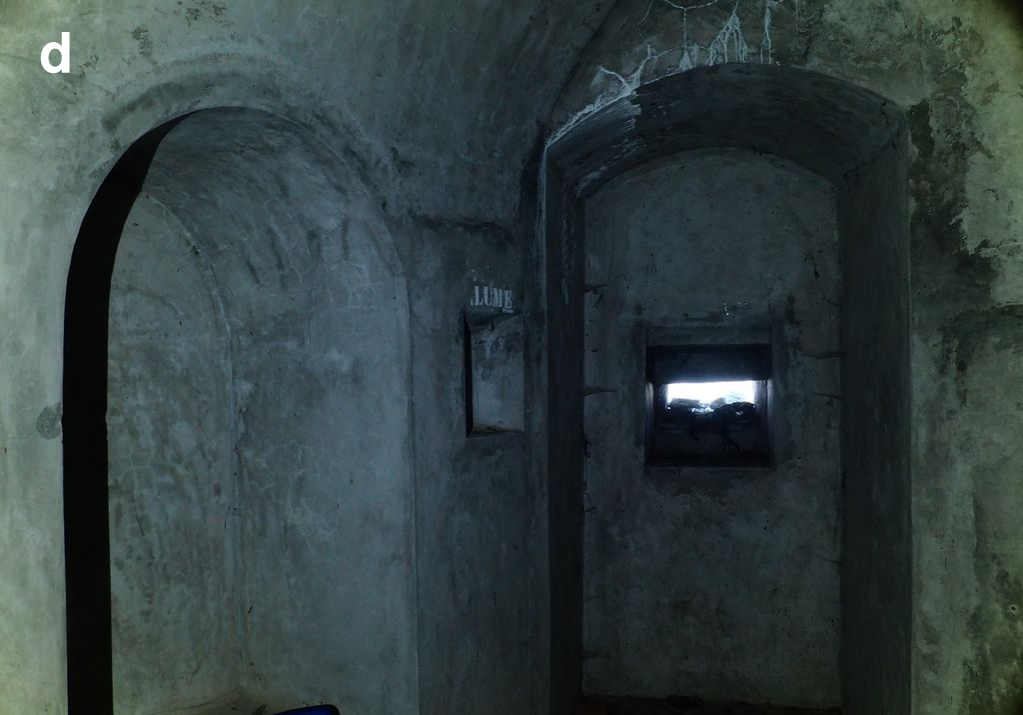
Military bunker, interior.
